# Cross-sectional and longitudinal associations between physical activity, sedentary behaviour and bone stiffness index across weight status in European children and adolescents

**DOI:** 10.1186/s12966-020-00956-1

**Published:** 2020-04-28

**Authors:** Lan Cheng, Hermann Pohlabeln, Wolfgang Ahrens, Fabio Lauria, Toomas Veidebaum, Charalambos Chadjigeorgiou, Dénes Molnár, Gabriele Eiben, Nathalie Michels, Luis A. Moreno, Angie S. Page, Yannis Pitsiladis, Antje Hebestreit, On behalf of the IDEFICS and I. Family Consortia

**Affiliations:** 1 Leibniz Institute for Prevention Research and Epidemiology – BIPS, Achterstraße 30, 28359 Bremen, Germany; 2 Faculty of Mathematics and Computer Science, University of Bremen, Bremen, Germany; 3 Institute of Food Sciences, National Research Council, Avellino, Italy; 4 Department of Chronic Diseases, National Institute for Health Development, Tallinn, Estonia; 5 Research and Education Institute of Child Health, Strovolos, Cyprus; 6 Department of Pediatrics, Medical School, University of Pécs, Pécs, Hungary; 7 Department of Public Health, School of Health Sciences, University of Skövde, Skövde, Sweden; 8 Department of Public Health, Faculty of Medicine and Health Sciences, Ghent University, 9000 Ghent, Belgium; 9 GENUD (Growth, Exercise, Nutrition and Development) Research Group, Instituto Agroalimentario de Aragón (IA2), Instituto de Investigación Sanitaria Aragón (IIS Aragón), Centro de Investigación Biomédica en Red Fisiopatología de la Obesidad y Nutrición (CIBERObn), University of Zaragoza, 50009 Zaragoza, Spain; 10 Centre for Exercise, Nutrition & Health Sciences, University of Bristol, Bristol, UK; 11 Collaborating Centre of Sports Medicine, University of Brighton, Brighton, UK

**Keywords:** Physical activity, Sedentary behaviour, Overweight, Bone stiffness index, Observational study

## Abstract

**Background:**

The associations between physical activity (PA), sedentary behaviour (SB) and bone health may be differentially affected by weight status during growth. This study aims to assess the cross-sectional and longitudinal associations between PA, SB and bone stiffness index (SI) in European children and adolescents, taking the weight status into consideration.

**Methods:**

Calcaneus SI was first measured by quantitative ultrasound among children aged 2–9 years old in 2007/08. It was measured again after 2 years in the IDEFICS study and after 6 years in the I. Family study. A sample of 2008 participants with time spent at sports clubs, watching TV and playing computer/games self-reported by questionnaire, and a subsample of 1037 participants with SB, light PA (LPA) and moderate-to-vigorous PA (MVPA) objectively measured using Actigraph accelerometers were included in the analyses. Weight status was defined as thin/normal and overweight/obese according to the extended International Obesity Task Force criteria. Linear mixed-effects models were used to estimate the cross-sectional and longitudinal associations between PA, SB and SI percentiles, stratified by weight status.

**Results:**

The cross-sectional association between weekly duration of watching TV and SI percentiles was negative in thin/normal weight group (β = − 0.35, *p* = 0.008). However, baseline weekly duration of watching TV (β = − 0.63, *p* = 0.021) and change after 2 years (β = − 0.63, *p* = 0.022) as well as the change in weekly duration of playing computer/games after 6 years (β = − 0.75, *p* = 0.019) were inversely associated with corresponding changes in SI percentiles in overweight/obese group. Change in time spent at sports clubs was positively associated with change in SI percentiles after 2 years (β = 1.28, *p* = 0.001), with comparable effect sizes across weight status. In the subsample with accelerometer data, we found a positive cross-sectional association between MVPA and SI percentiles in thin/normal weight group. Baseline MVPA predicted changes in SI percentiles after 2 and 6 years in all groups.

**Conclusions:**

Our results suggested the beneficial effect of PA on SI. However, the increasing durations of screen-based SB might be risk factors for SI development, especially in overweight/obese children and adolescents.

## Background

Bone strength is influenced by mass, architecture and density, while the trajectory of bone strength accrual persists up to the age of about 18 years until peak bone mass (PBM) is reached [1, 2]. Even though PBM is mainly explained by genetic determinants [3], it is also influenced by lifestyle-related factors such as mechanical loading, physical activity (PA), sedentary behaviour (SB) and nutrition [4, 5]. Further, PBM is an important predictor of osteoporosis in adults, due to the age-related bone loss that occurs over time [6]. Hence, in order to prevent fractures and osteoporosis in later life, it is important to initiate preventive measures during childhood and adolescence.

The positive osteogenic effect of PA, in particular weight-bearing exercises (WBEs), on bone strength seems to be irrefutable [7, 8]. However, despite these proven health benefits, the secular trend of PA shows a decrease among European children and adolescents, with most of them not meeting the World Health Organization (WHO) recommendations for PA [9–11]. Together with the decrease of PA, high levels of SB among this population group now constitute a serious public health concern [12]. In recent studies, the total duration of SB among 10- to 12-year-old European children was reported to be nearly 8 h per day [13], and they were also observed to spend more than 2 h per day in front of computer or TV screens [14]. The debate on the detrimental effects of SB on bone strength is, however, more controversial compared to beneficial effects of PA. Previous studies reported a negative [15] or null [16] association between the total duration of objectively measured SB using accelerometers and bone strength, while others suggested that self-reported screen-based SB such as using the internet [17], watching TV [18] and total screen time [19] may inversely influence bone mass. Currently, more studies are needed to combine self-reported data with objectively measured data when examining the short- and long-term effects of context-specific PA and SB on bone strength in young populations.

On one hand, sedentary lifestyles may be associated with poor bone health and are also linked to a higher risk of overweight and obesity in children and adolescents [20]. On the other hand, previous studies indicated that overweight or obese children have higher bone mass [21] or strength [22] compared to their normal weight peers, which, however, is in conflict with the unfavourable effects of sedentary lifestyles. In addition, being overweight has been reported to increase the risk for sedentary lifestyles [23], thereby leading to poor bone health. In view of these potential pathways, the associations between PA, SB and bone strength may be differentially influenced by overweight and obesity.

Understanding which specific dimensions of PA and SB influence the growing skeleton is crucial for the development of effective and sustainable strategies for increased bone strength. Particularly the role of weight status in these associations is still poorly understood. In an effort to fill this gap, information on the bone stiffness index (SI) measured using quantitative ultrasound (QUS) as a proxy indicator for bone strength has been repeatedly collected in a sample from the IDEFICS (Identification and prevention of dietary- and lifestyle- induced health effects in children and infants) and I. Family studies. Self-reported time spent at sports club, WBEs, watching TV and playing computer/games, as well as objectively measured SB, light PA (LPA) and moderate-to-vigorous PA (MVPA) were also collected to assess the cross-sectional and longitudinal associations of various kinds of PA and SB on SI in European children and adolescents across different weight statuses.

## Methods

### Study sample

The IDEFICS/I.Family study is the largest prospective child cohort in Europe with repeated measurements of anthropometric indicators, clinical examinations as well as extensive questionnaire-based information on socio-demographic factors, PA and nutrition [24, 25]. The first two waves of data collection occurred in the context of the IDEFICS study, which comprised 16,229 children from eight European countries (Belgium, Cyprus, Estonia, Germany, Hungary, Italy, Spain and Sweden), who were aged 2–9.9 years at baseline between September 2007 and May 2008. The second wave between September 2009 and May 2010 included a follow-up of 11,043 children from baseline and 2543 newly recruited children. The third wave was conducted in the context of the I. Family study between January 2013 and June 2014 with a follow-up of 7117 children from the original IDEFICS cohort and 2501 newly recruited children. All parents provided signed informed consent for their children prior to all exanimations. In addition, children younger than 12 years gave their oral consent and children above 12 years provided a signed simplified form of consent. Ethical approval for the study was obtained from the ethics committees for participating centres in each country.

### Inclusion and exclusion criteria

QUS measurements were obtained as an optional module in a subgroup of participants in the IDEFICS study, while in the I. Family study, QUS data were only available for five of the eight participating countries. We assumed that no substantial selection effects occurred since the reduced participation was mainly because of budgetary constraints and device feasibility. In order to simultaneously investigate cross-sectional and longitudinal associations between the exposures of interest and bone SI, we included 3422 children with baseline and at least one follow-up QUS measurements of both the left and right foot. In accordance with findings from a previous IDEFICS study on QUS measurement precision, there was a significant discrepancy in SI difference between the left and right foot across devices in each participating centre (unpublished data). In order to control for this discrepancy, 200 children whose QUS measurements had an SI difference between the left and right foot above 41 units (97th percentile, calculated based on 7612 repeated measurements in total) were excluded. A further 43 children who at baseline reported having medical conditions preventing participation in regular PA and/or known to influence bone metabolism were excluded [26].

**Fig. 1:**
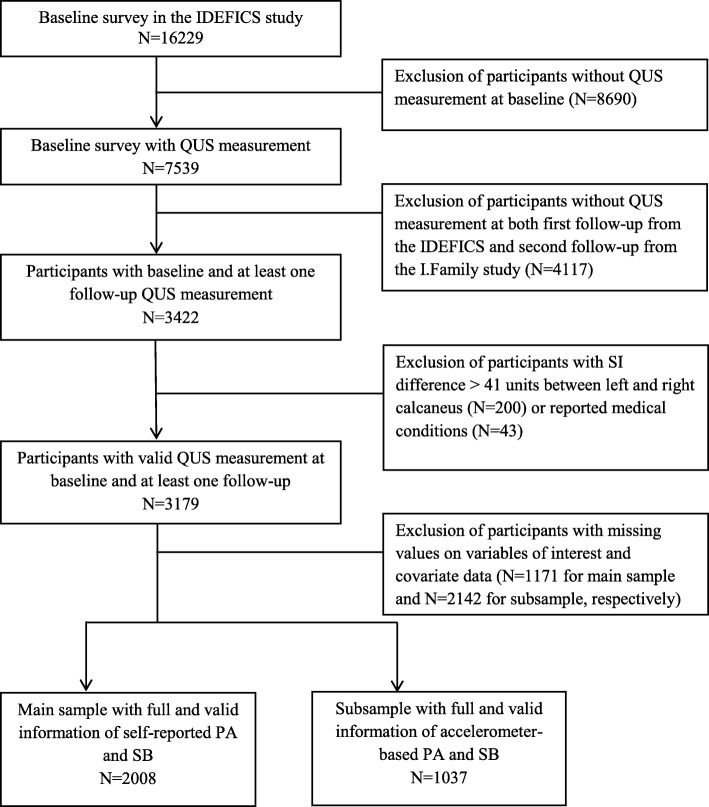
Flow diagram of children included in final analysis groups, baseline examination (IDEFICS): 2007/2008, two-year follow-up examination (IDEFICS): 2009/2010, six-year follow-up examination (I.Family): 2013/2014. *QUS* quantitative ultrasound, *PA* physical activity, *SB* sedentary behaviour

Finally, children without self-reported PA, screen-based SB or covariate data were excluded, leaving a total of 2008 participants for the full analysis. The mean age of the main sample was 6.14 years (SD = 1.80), 54.1% were boys and the proportions of low, medium and high familial socio-economic status (SES) were 10.4, 56.3 and 33.3%, respectively. A subsample of 1037 participants who provided objectively measured accelerometer-based SB, LPA and MVPA data was also analysed. The mean age of the subsample was 6.45 years (SD = 1.72), 50.4% were boys and the proportions of low, medium and high familial socio-economic status (SES) were 9.4, 60.3 and 30.3%, respectively. Compared with the original IDEFICS study sample, the children in both analytic samples were older (vs. 6.01 years, SD = 1.79) and more children had low (vs. 9.0%) and medium levels (vs. 50.3%) of SES. In addition, more boys were included in the main sample (vs. 50.8%). The inclusion and exclusion process of participants for the final analysis is summarised in Fig. 1. No children from Cyprus were included in the analysis as they did not fulfil any of the inclusion criteria.

### Bone stiffness index

QUS measurements on the left and right calcaneus were performed using Achilles Lunar Insight™ (GE Healthcare, Milwaukee, WI, USA). The parameters of speed of sound (SOS, m/s) and broadband ultrasound attenuation (BUA, dB/MHz) assessed by QUS devices reflect the velocity and attenuation of the ultrasound waves through the bone tissue, respectively. The SI value was estimated automatically by Lunar Achilles OsteoReport Software and reported as ‘unit’ according to the equation: SI = (0.67*BUA) + (0.28*SOS) – 420, with high SI values indicating better bone strength [27]. SOS, BUA and SI assessed by calcaneus QUS have been shown to be correlated with bone mineral content (BMC) and bone mineral density (BMD) assessed by dual energy X-ray absorptiometry (DXA) in children and adolescents in previous studies [28, 29]. There is also evidence suggesting that QUS devices could be used to estimate fracture risk and osteoporosis in childhood [27] and adulthood [30]. Compared to DXA, the main advantages of QUS devices are that they are non-radiating, quick and cost-effective, making them more suitable for large-scale epidemiological studies, particularly in healthy young populations. The SI value not only reflects bone density, it is also influenced by the architecture and elasticity of the bone tissue, which makes it possible to provide some structural information [27]. The reproducibility in each QUS device was tested on 91 children from the IDEFICS baseline; no differences were found in SI values between the three repeated measurements and between measurements at the left and right foot. The root-mean-square coefficients of variation (CV_RMS_) for the SI measurements were 7.2 and 9.2% on the left and right foot, respectively (unpublished data). In line with the study protocol, daily machine calibration was carried out during the entire study period and the measurements were taken by trained nurses according to the standard procedure [31]. Two different sizes of foot adapters were used to put the calcaneus in an appropriate position. The mean SI of the left and right calcaneus was calculated and used in the statistical analysis. The distribution of SI was assessed and age-, sex- and height-specific percentiles for SI values were calculated as outcomes [32].

### Anthropometric measurements

Height and weight were measured in light clothing without shoes. The former was measured to the nearest 0.1 cm using a standard clinical Seca 225 stadiometer (Seca, Hamburg, Germany) and the latter to the nearest 0.1 kg using a BC420 SMA scale (Tanita, Amsterdam, the Netherlands). The intra- and inter-observer reliability for height and weight were conducted in each centre, with CV% ranging from 0.2 to 1.0% in the IDEFICS [33] and I. Family study (unpublished data). In all study centres, trained nurses took the measurements following standardised procedures. For each child, age- and sex-specific z-scores of height and weight were determined using the LMS method by Cole et al. [34]. Body mass index (BMI, kg/m^2^) was calculated as body weight divided by squared body height, and weight status (thin/normal and overweight/obese) was classified at the 90th percentile (passing through the BMI of 25 at the age of 18) as recommended based on the extended International Obesity Task Force (IOTF) BMI criteria [35].

### Questionnaires

The questionnaires relating to lifestyle behaviours were answered by parents for young children up to 11 years old; they were self-reported for 12- to 15-year-old adolescents. The following information was collected for each child/adolescent: whether they were a member of a sports club and if so, 1) how many hours and minutes per week they spent there and 2) in what kind of sports they participated at the sports club. The time spent at sports clubs was calculated by adding the hours and minutes reported and expressed as hours per week (h/w). The variable WBE was based on all reported types of sports, classified according to the loads and categorised into moderate or high mechanical loads (ball games, gymnastics, dancing, skating, martial arts and athletics, etc.) and no or low mechanical loads (swimming, biking and horseback riding, etc.). In addition, information regarding the time usually spent watching TV/videos/DVDs and playing on a computer/game console on a normal weekday and weekend day was also collected. For both questions, six response categories were offered and converted into the following scoring system: not at all = 0, < 30 min = 1, < 1 h = 2, 1- < 2 h = 3, 2–3 h = 4, and > 3 h = 5. Each screen-based SB was calculated separately for weekdays and weekend days by adding the converted responses of the individual questions and expressed as hours per week (h/w). Weekly duration of watching TV was further classified into > 14 h/w and ≤ 14 h/w in accordance with international guidelines [36, 37] to investigate the benefit of fulfilling the guidelines on SI.

### Accelerometer data

PA and SB were objectively measured using Actigraph accelerometer devices (Actigraph, LLC, Pensacola, FL, USA) in a subsample of participants. Parents or legal guardians were asked to ensure that their child wore the accelerometer on the right hip and that it was only removed during water-based activities and bedtime. Data were collected in the vertical axis for three-axial accelerometer. In the IDEFICS study, either the GT1M or ActiTrainer was used; the sensor units of both models are identical. In previous validation studies, both types of accelerometers have been observed to measure comparable MPA, LPA and MVPA levels [38]. However, the outputs of counts per minute (cpm) [39] and low PA levels (e.g. LPA and walking) [38] for ActiTrainer were lower than other Actigraph models, thus care should be taken when interpreting these results. In the I. Family study, either the GT1M or GT3x + was used, the comparability of the vertical axis outputs for the GT3X and GT1M has also been proven in previous studies [40, 41]. The participants were requested to wear the accelerometer for at least 3 days including one weekend day in the IDEFICS study and for 7 days in the I. Family study. Participants were included in the analyses only if they had at least 6 h of data per day and three accelerometer measurement days. Further, any periods containing 20 min or more of consecutive zero counts were removed as non-wearing time. All accelerometer recordings were integrated over 60s epochs and the intensity levels were classified as SB (≤100 cpm), LPA (> 100- < 2296 cpm) and MVPA (≥2296 cpm) according to the cut-off points suggested by Evenson et al. [42]. More details on processing of accelerometer data in the IDEFICS/I.Family study have been published elsewhere [9, 43]. In the present study, total durations of objectively measured SB and LPA were expressed as hours per day (h/d). In order to better interpret the regression coefficients, the unit of objectively measured MVPA was converted to 10 min per day (10 min/d) according to previous studies, reporting that every additional 10 min/d of MVPA was associated with increases of bone health indicators in children [26, 44]. We further considered the variable objectively measured MVPA as a dichotomised instead of a continuous variable. According to WHO recommended levels of PA for children and adolescents aged 5–17 years old, daily duration of objectively measured MVPA ≥1 h/d was regarded as adhering to the guideline [45].

### Confounding variables

Sex, age and questions regarding the familial SES of participants were reported by parents. SES was assessed based on the highest educational level of parents according to the International Standard Classification of Education (ISCED) and categorised into low (ISCED 0,1,2), medium (ISCED 3,4) and high (ISCED 5,6) [46]. The voice change of boys and the first menstrual period of girls from the age of 8 years old were collected in the I. Family study as a proxy for pubertal development and further categorised into pre-pubertal and pubertal in the present study. Both indicators have been widely used to assess maturation in previous epidemiological studies, suggesting that changes in the male voice often occur between Tanner stages 3 and 4 [47, 48], which is the comparable onset age of menarche in females [49]. The variance between countries was also considered. Further, sunlight exposure as the most important source for vitamin D synthesis was also taken into consideration [50], calculated by mean daylight duration for each examination month in each location based on astronomical tables [26].

### Statistical analyses

All analyses were performed using SAS software (V9.3; SAS Institute Inc., Cary, North Carolina, USA). The changes in continuous variables were determined by calculating the differences between follow-up after 2 or 6 years and baseline values. Descriptive statistics, e.g. means, standard deviations (SD), and frequencies for baseline and changes of each variable were conducted and stratified by weight status (thin/normal and overweight/obese) in each survey. Differences for continuous variables were compared using t tests, and chi-square tests were used for categorical variables.

Linear mixed-effects models were used to estimate the cross-sectional and longitudinal associations between PA, SB and SI percentiles, with country as a random effect (at the level of the intercept). To avoid getting associations that are irrelevantly statistically significant, for instance simply due to the large sample size or to multiple testing, a more stringent criterion for statistical significance (α = 0.01) was chosen. Regression coefficients (β) and 99%-confidence intervals (99%CI) were estimated in all models. The cross-sectional analyses were based on the data from baseline and the outcome was baseline SI percentiles. Weekly duration of watching TV, playing computer/games and sports club activities as well as WBE were taken into consideration as exposures and adjusted for sex, age, SES, daylight duration, weight and height z-scores. In the longitudinal analyses, the outcomes were the changes in SI percentiles after 2 or 6 years, with taking the baseline and changes in exposures into consideration. In addition to the confounding factors described above, we also included baseline SI percentiles and pubertal status in the longitudinal models. Based on the same analytical approach, objectively measured SB, LPA and MVPA were considered in subgroup analyses and presented separately. All analyses were performed in the whole group and then further stratified by thin/normal and overweight/obese groups. Differences in the association between each exposure of interest and corresponding outcome across weight status were further tested by interactive terms in the whole group models, however, they were not considered in the final analyses since no statistically significant interactions were observed.

## Results

### Descriptive characteristics of study population

As summarised in Table 1, the baseline proportions of overweight/obese children in the main sample (*n* = 2008) and subsample with accelerometer data (*n* = 1037) were 19.0 and 19.7%, respectively. At individual country-level, Italy had the highest proportion of overweight/obese children and Belgium the lowest. Regarding SI, in the main sample, the means of SI percentiles were 43.92 ± 27.84 in the thin/normal weight group and 41.36 ± 25.76 in the overweight/obese group at baseline with an increase for both groups during the two-year and six-year follow-up periods (Table 2). Increasing trends in the weekly duration of watching TV, playing computer/games and time spent at sports clubs were also observed after 2 and 6 years. In the subsample with accelerometer data, there was a trend of increasing SB while LPA and MVPA slightly decreased over time.

**Table 1:** Demographic characteristics of the study population, stratified by weight status

	Main sample ^a^		Subsample ^b^	
	Thin/normal weight	Overweight/obese	Thin/normal weight	Overweight/obese
	(*N* = 1627)	(*N* = 381)	(*N* = 833)	(*N* = 204)
Age (Mean, SD) ^c^	6.02 (1.81)	6.64 (1.66)	6.35 (1.73)	6.83 (1.63)
Sex (N, %) ^c^				
Boys	911 (83.8)	176 (16.2)	435 (83.2)	88 (16.8)
Girls	716 (77.7)	205 (22.3)	398 (77.4)	116 (22.6)
Family socio-economic status (N, %) ^c^				
Low	142 (68.3)	66 (31.7)	69 (70.4)	29 (29.6)
Medium	893 (79.0)	238 (21.0)	500 (80.0)	125 (20.0)
High	592 (88.5)	77 (11.5)	264 (84.1)	50 (15.9)
Pubertal status (N, %) ^d^				
Pre or early pubertal	343 (78.3)	95 (21.7)	174 (78.7)	47 (21.3)
Pubertal	180 (64.8)	98 (35.4)	128 (72.3)	49 (27.7)
Country (N, %) ^c^				
Belgium	278 (93.0)	21 (7.0)	68 (93.1)	5 (6.9)
Estonia	274 (84.3)	51 (15.7)	183 (89.3)	22 (10.7)
Germany	472 (85.2)	82 (14.8)	310 (85.4)	53 (14.6)
Hungary	112 (88.9)	14 (11.1)	45 (83.3)	9 (16.7)
Italy	259 (60.7)	168 (39.3)	85 (52.5)	77 (47.5)
Spain	116 (81.1)	27 (18.9)	124 (78.5)	34 (21.5)
Sweden	116 (86.6)	18 (13.4)	18 (81.8)	4 (18.2)

^a^Main sample included participants with full information of self-reported physical activity and sedentary behaviour as well as co-variables. ^b^Subsample included the participants with full information of accelerometer data as well as co-variables. ^c^Data from baseline survey; ^d^Data from six-year follow-up survey

**Table 2:** Baseline and changes of measurements over two-year and six-year follow-up

	Baseline		Two-year changes		Six-year changes	
	Thin/normal weight	Overweight/obese	Thin/normal weight	Overweight/obese	Thin/normal weight	Overweight/obese
	(N = 1627 ^a^/833 ^b^)	(N = 381 ^a^/204 ^b^)	(*N* = 1273 ^a^/633 ^b^)	(*N* = 380 ^a^/206 ^b^)	(*N* = 523 ^a^/302 ^b^)	(*N* = 193 ^a^/96 ^b^)
Anthropometric measures (Mean, SD) ^c^						
Percentiles of bone stiffness index	43.92 (27.84)	41.36 (25.76)	3.86 (30.17)	4.60 (25.55)	8.08 (34.59) ^*^	18.88 (33.46) ^*^
Height z-score	0.37 (1.00) ^*^	0.87 (1.02) ^*^	0.04 (0.40)	0.06 (0.39)	0.20 (0.60) ^*^	0.05 (0.68) ^*^
Weight z-score	0.07 (0.87) ^*^	2.00 (0.78) ^*^	0.03 (0.38) ^*^	0.19 (0.52) ^*^	0.21 (0.66) ^*^	0.43 (0.87) ^*^
Reported healthy behaviour						
Watching TV/video/DVD (hours/week, Mean, SD) ^c^	8.67 (5.00) ^*^	9.74 (5.32) ^*^	0.62 (4.57)	0.71 (5.13)	2.05 (6.41)	3.09 (7.57)
Playing computer/games (hours/week, Mean, SD) ^c^	2.34 (3.39)	2.69 (3.58)	1.38 (3.58) ^*^	2.17 (4.66) ^*^	2.84 (5.83)	3.63 (7.16)
Sports clubs (hours/week, Mean, SD) ^c^	1.13 (1.60)	1.32 (1.63)	0.72 (1.77)	0.56 (1.89)	1.41 (2.56)	0.90 (2.67)
Weight bearing sports (N, %) ^d^						
Moderate or high mechanical loads	752 (79.5)	194 (20.5)	866 (76.5)	266 (23.5)	396 (73.5)	143 (26.5)
No or low mechanical loads	875 (82.4)	187 (17.6)	407 (78.1)	114 (21.9)	127 (71.8)	50 (28.2)
Accelerometer data (Mean, SD)						
Sedentary time (hours/day) ^c^	4.46 (1.21) ^*^	4.72 (1.26) ^*^	0.70 (1.39)	0.61 (1.42)	2.31 (1.53)	2.41 (1.59)
Light physical activity (hours/day) ^c^	6.37 (1.00)	6.39 (1.10)	−0.46 (1.14)	−0.57 (1.23)	−1.47 (1.28)	− 1.56 (1.29)
Moderate-to-vigorous physical activity (10 min/day) ^c^	4.28 (2.21) ^*^	3.57 (1.93) ^*^	−0.23 (2.43)	− 0.45 (1.96)	0.10 (2.61)	− 0.50 (2.72)

^a^ Sample size with full information of self-reported physical activity and sedentary behaviour as well as co-variables. ^b^ Sample size with full information of accelerometer data as well as co-variables. ^c^ Changes of values were the differences between follow-up and baseline measurements. ^d^ Changes of values were the percentages of reported moderate or high mechanical loads at baseline or follow-up, and no or low mechanical loads in both waves, respectively. * *p* < 0.01

### Cross-sectional associations between SB, PA and SI percentiles

No statistically significant associations between self-reported and objectively measured SB, PA and SI were observed in the whole group at baseline. However, in thin/ normal weight group, weekly duration of watching TV was inversely associated with SI percentiles (β = − 0.35, *p* = 0.008), while daily duration of objectively measured MVPA was positively associated with SI percentiles (β = 1.18, *p* = 0.008). Opposite but not statistically significant associations were observed for the overweight/obese group where SI percentiles were positively associated with the weekly duration of watching TV (β = 0.03, *p* = 0.906) while inversely associated with the daily duration of objectively measured MVPA (β = − 0.23, *p* = 0.807) (Table 3).

**Table 3:** Cross-sectional associations between sedentary behaviour, physical activity and bone stiffness index percentiles at baseline

	Whole group		Thin/normal weight group		Overweight/obese group	
	β (99%CI)	*p*-value	β (99%CI)	p-value	β (99%CI)	p-value
*Main sample (self-reported data, N = 2008)*						
Watching TV/video/DVD (hours/week)	− 0.23(− 0.53,0.06)	0.044	− 0.35(− 0.69,-0.01)	0.008	0.03(− 0.60,0.66)	0.906
Playing computer/games (hours/week)	− 0.004(− 0.49,0.48)	0.984	0.03(− 0.52,0.58)	0.883	0.03(− 0.96,1.01)	0.943
Sports clubs (hours/week)	1.00(− 0.40,2.39)	0.066	0.50(− 1.03,2.04)	0.398	2.68(− 0.58,5.95)	0.034
Weight bearing sports						
Moderate or high mechanical loads vs. No or low mechanical loads (reference)	− 1.80(−6.37,2.77)	0.310	− 0.13(−5.16,4.91)	0.948	−8.05(− 18.68,2.59)	0.051
*Subsample (accelerometer data, N = 1037)*						
Sedentary time (hours/day)	− 0.11(− 2.11,1.90)	0.891	1.01(− 1.29,3.31)	0.256	−3.85(−7.82,0.13)	0.013
Light physical activity (hours/day)	−0.94(− 3.06,1.19)	0.255	−1.46(− 3.90,0.99)	0.124	− 0.22(− 4.50,4.05)	0.893
Moderate-to-vigorous physical activity (10 min/day)	0.70(− 0.32,1.73)	0.077	1.18 (0.03,2.33)	0.008	− 0.23(− 2.62,2.17)	0.807

Adjusted for baseline age, sex, socio-economic status, daylight, height and weight z-score, country as a random effect

### Longitudinal effects of SB and PA on changes in SI percentiles

In the whole group, change in time spent at sports clubs was positively associated with change in SI percentiles after 2 years (β = 1.28, *p* = 0.001); objectively measured MVPA at baseline was a strong predictor for change in SI percentiles (β = 2.77, *p* < 0.001). Similar effect sizes were observed after stratifying by weight status although the findings for overweight/obese group were not statistically significant. In contrast, weekly duration of watching TV at baseline (β = − 0.63, *p* = 0.021) and change after 2 years (β = − 0.63, *p* = 0.022) were inversely associated with change in SI percentiles only in overweight/obese group (Table 4).

**Table 4:** Longitudinal associations between sedentary behaviour, physical activity and bone stiffness index percentiles after 2 years

	Whole group		Thin/normal weight group		Overweight/obese group	
	β (99%CI)	*p*-value	β (99%CI)	p-value	β (99%CI)	p-value
*Main sample (self-reported data,, N = 1653)*						
Baseline watching TV/video/DVD (hours/week)	0.06(− 0.33,0.44)	0.712	0.28(− 0.18,0.74)	0.118	− 0.63(− 1.34,0.07)	0.021
Baseline playing computer/games (hours/week)	0.02(− 0.57,0.60)	0.943	− 0.12(− 0.81,0.57)	0.651	0.42(− 0.67,1.50)	0.318
Baseline sports clubs (hours/week)	1.07(− 0.23,2.36)	0.034	0.96(− 0.54,2.46)	0.098	1.03(− 1.54,3.60)	0.299
Change of watching TV/video/DVD (hours/week)	0.11(− 0.28,0.49)	0.468	0.36(−0.10,0.81)	0.042	−0.63(−1.35,0.08)	0.022
Change of playing computer/games (hours/week)	0.09(−0.35,0.54)	0.593	0.09(− 0.47,0.64)	0.688	0.32(− 0.41,1.04)	0.258
Change of sports clubs (hours/week)	1.28 (0.30,2.26)	0.001	1.29 (0.10,2.48)	0.005	1.04(−0.63,2.70)	0.110
Weight bearing sports						
Moderate or high mechanical loads vs. No or low mechanical loads (reference)	−0.69(−4.99,3.61)	0.678	0.32(− 4.76,5.41)	0.870	−3.13(−11.14,4.89)	0.313
*Subsample (accelerometer data, N = 839)*						
Baseline sedentary time (hours/day)	0.60(−1.77,2.96)	0.516	0.99(−1.86,3.85)	0.369	−0.39(−4.65,3.86)	0.811
Baseline light physical activity (hours/day)	−0.81(−3.50,1.89)	0.439	−1.60(−4.93,1.73)	0.214	0.16(−4.46,4.78)	0.927
Baseline moderate-to-vigorous physical activity (10 min/day)	2.77 (1.50,4.05)	< 0.001	2.97 (1.53,4.42)	< 0.001	2.85(−0.12,5.81)	0.013
Change of sedentary time (hours/day)	−0.85(−2.58,0.88)	0.205	−1.19(−3.25,0.87)	0.135	0.10(−3.09,3.29)	0.935
Change of light physical activity (hours/day)	−0.94(−3.03,1.16)	0.249	−1.45(−3.99,1.09)	0.140	0.42(−3.26,4.10)	0.768
Change of moderate-to-vigorous physical activity (10 min/day)	1.05(−0.09,2.18)	0.018	0.89(−0.39,2.16)	0.073	1.76(−0.88,4.40)	0.084

Adjusted for baseline age, sex, socio-economic status, daylight, bone stiffness index percentiles, height and weight z-scores, country as a random effect

Regarding the six-year follow-up, a statistically significant positive association between change in time spent at sports clubs and corresponding change in SI percentiles was observed only for the thin/normal weight group. As observed after 2 years, objectively measured MVPA at baseline also predicted change in SI percentiles after 6 years (β = 3.67, *p* < 0.001). In contrast to the slight effect of watching TV over the two-year period in overweight/obese group, we observed that six-year change in duration of playing computer/games was negatively associated with six-year change in SI percentiles (β = − 0.75, *p* = 0.019) (Table 5).

**Table 5:** Longitudinal associations between sedentary behaviour, physical activity and bone stiffness index percentiles after 6 years

	Whole group		Thin/normal weight group		Overweight/obese group	
	β (99%CI)	p-value	β (99%CI)	p-value	β (99%CI)	p-value
*Main sample (self-reported data, N = 716)*						
Baseline watching TV/video/DVD (hours/week)	− 0.18(− 0.80,0.44)	0.444	−0.18(− 0.93,0.57)	0.531	−0.36(−1.50,0.77)	0.404
Baseline playing computer/games (hours/week)	0.24(−0.59,1.08)	0.448	0.33(−0.73,1.39)	0.420	−0.16(−1.51,1.20)	0.766
Baseline sports clubs (hours/week)	1.79(−0.16,3.74)	0.018	1.99(−0.29,4.27)	0.024	0.59(−3.20,4.38)	0.686
Change of watching TV/video/DVD (hours/week)	−0.16(− 0.61,0.29)	0.362	− 0.20(− 0.74,0.34)	0.335	−0.10(− 0.93,0.72)	0.744
Change of playing computer/games (hours/week)	−0.13(− 0.59,0.33)	0.472	0.11(− 0.45,0.67)	0.617	−0.75(−1.58,0.07)	0.019
Change of sports clubs (hours/week)	1.01(−0.10,2.12)	0.020	1.54 (0.23,2.85)	0.002	−0.46(−2.64,1.73)	0.588
Weight bearing sports						
Moderate or high mechanical loads vs. No or low mechanical loads (reference)	4.47(−2.29,11.22)	0.088	2.48(−5.39,10.36)	0.416	11.26(−1.69,24.22)	0.025
*Subsample (accelerometer data, N = 398)*						
Baseline sedentary time (hours/day)	−0.44(−4.00,3.13)	0.751	0.08(−4.19,4.35)	0.961	1.03(−5.82,7.88)	0.693
Baseline light physical activity (hours/day)	−2.70(−7.01,1.62)	0.106	−1.68(−6.76,3.41)	0.393	−5.13 (12.46,2.19)	0.068
Baseline moderate-to-vigorous physical activity (10 min/day)	3.67 (1.55,5.79)	< 0.001	3.49 (1.03,5.95)	< 0.001	4.94 (0.92,8.97)	0.002
Change of sedentary time (hours/day)	−0.18(−2.55,2.18)	0.840	0.16(−2.69,3.01)	0.884	−1.61(−5.51,2.28)	0.277
Change of light physical activity (hours/day)	−0.53(−3.78,2.71)	0.670	0.11(−3.70,3.92)	0.938	−2.18(−7.82,3.45)	0.309
Change of moderate-to-vigorous physical activity (10 min/day)	1.53(−0.04,3.10)	0.012	1.74(−0.10,3.59)	0.015	0.87(−2.09,3.83)	0.441

Adjusted for baseline age, sex, socio-economic status, daylight, bone stiffness index percentiles, height and weight z-scores and puberty at six-year follow-up, country as a random effect

### Effects of adherence to international PA and SB guidelines on SI percentiles

At baseline, 17.3% of the participants with accelerometer data adhered to the PA guideline of at least 1 h/d of objectively measured MVPA. When looking at the longitudinal data, only 6.3% adhered to the guideline at both baseline and two-year follow-up and 4.0% at both baseline and six-year follow-up. For participants who fulfilled the PA guideline at both time points, there was a higher increase of SI percentiles than for their counterparts with 10.39 units (*p* = 0.002) and 12.68 units (*p* = 0.050) over the two-year and six-year periods, respectively. Meanwhile, 88.8% of participants adhered to the screen time guideline of watching TV for no more than 14 h/w at baseline, 80.6% adhered to the guidelines at both baseline and two-year follow-up and 69.6% at both baseline and six-year follow-up. However, no associations were found between screen time guidelines and SI percentiles.

## Discussion

Our results highlighted the importance of objectively measured MVPA at baseline for the development of a healthy SI over two-year and six-year follow-up. These findings were robust and the effect sizes were consistent across weight statuses. We further demonstrated the benefit of adherence to established PA guidelines on long-term SI gain in children and adolescents, with those participating in objectively measured MVPA for at least 1 h per day having higher SI increases than their counterparts. These objectively measured results were supported by the comparable, albeit weak positive associations between self-reported time spent at sports clubs and changes in SI percentiles at two-year as well as six-year follow-ups. Regarding our assumption that being overweight/obese may be an important confounder, we observed controversial associations of screen-based SB with SI in the different weight strata. In general, the inversely cross-sectional associations between watching TV and SI were more pronounced in thin/normal weight children and adolescents than in overweight/obese ones. Nonetheless, in the longitudinal data, durations of specific screen-based SB were observed to be negatively associated with SI changes only in overweight/obese participants at the two-year and six-year follow-ups.

Even though the beneficial osteogenic effect of PA on bone mass accrual has already been well described in previous observational studies, most of the existing evidence so far mainly focused on BMC and/or BMD [51, 52]. Only a few cross-sectional studies have examined the associations between PA and QUS bone parameters. For example, Robinson et al. [53] demonstrated that time spent on moderate-to-high impact activities positively related to calcaneus SI in adolescent girls. Zulfarina et al. [54] reported that PA level, in terms of metabolic equivalent-minutes per week, was positively associated with three QUS parameters (i.e. BUA, SOS and SI) in adolescents. However, the PA levels in previous studies were mainly measured using different self-reported questionnaires, rendering it difficult to compare the results. Moreover, little is known about the optimal dose and intensity of PA and their sustainable effects on bone strength during growth. In our previous case-control study that was embedded in the IDEFICS study, we found that 30 min of objectively measured MVPA per day was not sufficient for an optimal SI [55]. A longitudinal study suggested that children in the upper quartile of objectively measured MVPA (approximated 1 h/d) had about 4 to 13% greater HR-pQCT-measured bone parameters at distal tibia compared to their peers in the lowest quartile (approximated 0.5 h/d) [56]. Our findings not only demonstrate that objectively measured MVPA rather than LPA using accelerometers is an important predictor of bone strength across weight strata, but also support the current opinion that adherence to the WHO recommendations for MVPA has a positive impact on bone strength. A recent systematic review suggested that more than 80% of adolescents had insufficient physical activity globally illustrating the urgent need for further effective policies and intervention strategies in order to obtain optimal bone strength in children and adolescents [57].

Regarding self-reported PA at baseline, less than half of the parents reported that their children participated in a sports club. The average time spent at sports clubs was given as approximately 1 h per week. This indeed depicts reality, as children of that age do not commonly take part in sports club activities. Our finding of change in time spent at sports clubs rather than baseline time being more strongly related to change in SI was hence to be expected, as the participants were then 2 and 6 years older at the respective follow-ups. While no association between self-reported WBE and SI was observed in our study, the osteogenic effect of WBE has been reported in a review regarding school-based intervention programs, however, focusing mainly on jumping exercises [58]. Bone strength is thought to be less sensitive to light and moderate WBE among growing individuals [59]. As we did not have data describing the intensity of WBE in our study this may possibly explain why our results were non-significant. Further, as the self-reported sports club activities did not include WBE during leisure time, the effect of WBE on SI may have been underestimated.

Notably, the cross-sectional associations we observed did not match the longitudinal associations across weight strata, especially in self-reported screen-based SB. A possible explanation could be that the deleterious effects of watching TV on SI were covered by the stimulating effect of the mechanical loading exerted by weight status, which increased along with more screen time [60]. However, after relatively long-term exposure to screen-based SB, less SI gain still could occur in overweight/obese children. Potential direct and indirect mechanisms of weight status may influence the longitudinal relationship between screen-based SB and SI development. On the one hand, being sedentary may disrupt the bone formation-resorption balance due to lack of mechanical loading [61]. This detrimental effect may be stronger in overweight/obese individuals since they lose more mechanical loading exerted by body weight. On the other hand, overweight/obese children are also likely to spend more time watching TV or playing computer/games [62], eventually resulting in reduced SI. A similar observation was made in our data. Moreover, detrimental effects of screen-based SB appeared to be pattern-specific over time, with the durations of watching TV and playing computer/games observed to be inversely associated with SI gain at two-year and six-year follow-up, respectively. Although watching TV, the predominantly measured screen-based SB, represents the largest amount of screen-based SB for most children, recent studies suggest that computer use has increased dramatically over the years and has even replaced time spent watching TV, especially in adolescents [63, 64]. Marco et al. [17] also reported that non-study internet use rather than watching TV was negatively associated with whole body BMC in male adolescents. Our results are in line with this behavioural transition from childhood to adolescence and demonstrate that playing computer/games might present a higher risk factor for bone strength than watching TV as children get older.

The relationship between objectively measured SB and bone health during growth is still inconclusive. Results of a recent systematic review indicated the presence of a minor association between total SB and bone outcomes of the lower extremities in youth [65]. In the British Columbia Healthy Bones Study III cohort (HBSII), no associations were found between screen-based SB, total SB and bone architecture and strength in 9- to 20-year-old subjects at baseline [16], but total duration of objectively measured SB was found to be a negatively independent predictor in longitudinal analyses based on four annual follow-ups [56]. In contrast, we did not observe any longitudinal relationships between total duration of objectively measured SB and SI, except for a small cross-sectional inverse association in overweight/obese children. In our previous IDEFICS study with a larger cross-sectional sample, we found that total duration of objectively measured SB was negatively associated with SI in preschool and school children [26]. As only a fraction of the subgroup of participants in the present study who had accelerometer data could be linked in longitudinal data, we believe that the associations we detected did not reach the significance threshold due to the small sample size. Nevertheless, from our investigation, it still can be concluded that SB operationalised as screen time might be a valuable predictor of bone strength. However, the optimal dose of SB as well as of specific screen-based SB on bone strength needs to be further investigated in longitudinal studies and interventions.

Our study has several strengths and limitations. To our best knowledge, this is the first longitudinal study to present the associations between SB, PA and QUS parameters using repeatedly measured data among European children and adolescents. Moreover, in addition to self-reported questionnaires, we investigated SB and PA with objective measurements in a relatively large subsample and thereby acquired more precise information regarding the intensity and quantification of activity levels. Additionally, we were able to identify differential associations of SI across weight strata, which helped provide better insight into the role of weight status in these associations. While we were able to collect some objective data, the fact that the data was only available for a subsample whose size diminished considerably at each follow-up is a limitation that most likely led to the lack of statistical power, and our results may not be generalised for the whole population. However, as the subsample’s baseline mean of SI percentiles (41.19 ± 26.58) and rate of overweight/obesity (19.7%) were comparable to that of the main sample (43.44 ± 27.47 and 19.0%, respectively), we believe that this reduces the potential for bias. Second, the imprecision of the self-reported WBE (only based on sports club activities) may have led to underestimations regarding cross-sectional and longitudinal effects of WBE on SI. Third, the comparability of ActiTrainer with other Actigraph accelerometers in previous validation studies is still inconclusive. However, we additionally included a confounder to account for different measures induced by the use of GT1M and ActiTrainer, which did not change our final results. Therefore, we are convinced that our data collection by different types of accelerometers provides comparable PA values. Finally, we did not include nutritional variables such as calcium intake. Instead, we considered weekly frequency of milk and dairy products consumption from a food frequency questionnaire as a proxy. Since we did not observe an influential effect on our final results, we did not consider this variable further to avoid having to exclude more participants, which would have reduced the sample size considerably.

## Conclusions

In summary, our results demonstrated that objectively measured MVPA is an important predictor of bone strength across weight strata. Meeting the MVPA recommendation of 1 h per day maintained the beneficial effect on bone strength during the six-year observational period. On the other hand, the increasing durations of screen-based SB might be risk factors for SI development, especially in overweight/obese participants. Finally, bone health improving interventions should promote high intensive exercises and also focus on the reduction of screen-based SB, particularly when targeting overweight/obese individuals.

## Data Availability

The datasets generated and analysed during the current study are not publicly available because this study is based on highly sensitive data collected in young children. But interested researchers can contact the IDEFICS and I. Family consortia (http://www.ideficsstudy.eu/Idefics/ and http://www.ifamilystudy.eu/) to discuss possibilities for data access.

## Abbreviations

PBM: Peak bone mass
PA: Physical activity
SB: Sedentary behaviour
WBE: Weight-bearing exercises
WHO: World Health Organization
LPA: Light physical activity
MVPA: Moderate-to-vigorous physical activity
QUS: Quantitative ultrasound
SI: Stiffness index
BUA: Broadband ultrasound attenuation
SOS: Speed of sound
BMC: Bone mineral content
BMD: Bone mineral density
DXA: Dual energy X-ray absorptiometry
BMI: Body mass index
IOTF: International Obesity Task Force
CPM: Counts per minute
SES: Socio-economic status
ISCED: International Standard Classification of Education
SD: Standard deviation
CI: Confidence interval
